# A novel molecular typing method of *Mycobacteria* based on DNA barcoding visualization

**DOI:** 10.1186/2043-9113-4-4

**Published:** 2014-02-20

**Authors:** Bin Liu, Xiaotian Zhang, Honglan Huang, Ying Zhang, Fengfeng Zhou, Guoqing Wang

**Affiliations:** 1 Cardiovascular disease center, First Hospital of Jilin University, Changchun, Jilin, China; 2 Department of Pathogenobiology, Basic Medical College of Jilin University, Changchun, Jilin, China; 3 Shenzhen Institutes of Advanced Technology, and The Key Laboratory for Health Informatics, Chinese Academy of Sciences, Shenzhen, Guangdong, China

**Keywords:** *Mycobacterium*, Molecular typing, Typing biomarker, Bioinformatics, Differential diagnosis of *mycobacteria*

## Abstract

Different subtypes of *Mycobacterium tuberculosis* (MTB) may induce diverse severe human infections, and some of their symptoms are similar to other pathogenes, *e.g. Nontuberculosis mycobacteria* (NTM). So determination of *mycobacterium* subtypes facilitates the effective control of MTB infection and proliferation. This study exploits a novel DNA barcoding visualization method for molecular typing of 17 *mycobacteria* genomes published in the NCBI prokaryotic genome database. Three *mycobacterium* genes (Rv0279c, Rv3508 and Rv3514) from the PE/PPE family of MT Band were detected to best represent the inter-strain pathogenetic variations. An accurate and fast MTB substrain typing method was proposed based on the combination of the aforementioned three biomarker genes and the 16S rRNA gene. The protocol of establishing a bacterial substrain typing system used in this study may also be applied to the other pathogenes.

## Introduction

*Nontuberculosis mycobacteria* (NTM) are a diverse group of organisms that are ubiquitous in both natural and manmade environments [1]. Though less notorious than *Mycobacterium tuberculosis* (MTB), NTM infections are also of clinical significance and have been associated with worldwide outbreaks in the past. A previous study showed that the clinical symptoms and iconography representation of NTM were similar to MTB making it difficult to differentiate between the two diseases. Furthermore, treatment is also more difficult because most of NTM are naturally resistant to anti-tuberculosis drugs [2]. Thus, there is an urgent clinical need for tools that would enable accurate differentiation of MTB from NTM-induced disease. Since the genomes of different *mycobacteria* have been sequenced, it is now possible for us to generate a novel DNA barcoding technology for genotyping of *mycobacteria*.

Clinically, *mycobacteria* were traditionally characterized based on acid-fastness, smear and culture morphology, growth rate, pigment production and various biochemical tests [3]. These parameters provide a useful tool to aid MTB diagnosis. However, a higher degree of differentiation, including the ability to distinguish between species and subspecies has become a requirement in both epidemiological and clinical settings. Thus, molecular based techniques could allow faster species identification and phylogenetic analyses. There are numerous published methods for *mycobacteria* genotyping, including insertion sequence (IS) 6110 restriction fragment length polymorphism (RFLP) analysis, PCR-based techniques, such as mycobacterial interspersed repetitive unit-variable number of tandem repeat (MIRU-VNTR) analysis, and so on. Despite the availability of all of these techniques, IS 6110-RELP has fallen out of favor because of cost and high quantities of purified genomic DNA requirements. Moreover, it is not applicable for trains with low copy numbers of IS6110 [4]. Although MIRU-VNTR is accurate and effective in genotyping, however, to date, selection or choice of the mycobacterium-typing region is still problematic with considerable variation in the genotyping efficiency of different regions and lack of accuracy and uniformity [5]. The underlying reasons for this could be ascribed to i) high conservation of *mycobacteria* nucleotide sequences and the low information content contained in simple sequence features; and ii) low distinguishability for the codon usage bias among different species, specific nucleotide distance and other biological characteristics.

We have previously shown that the frequency spectrum of each k-mer nucleotide string (K, 1 < K <6) within the region of equal length fragments in the microbial genome was consistent [6]. It is therefore possible to obtain a barcode-like visual annotation (Barcode image) of a genome by constructing a digital and graphical process for the array matrix of the frequency spectrum. According to this hypothesis, any microbial genome can be represented as a unique barcode image. A genome barcode could carry all the genetic information in a given genome and exhibit a one-to-one correspondence with the genome sequence. Genome barcodes not only provide a useful tool to visualize any given genome, but also allows us to easily compare different genomes by calculating the whole genome k-mer average frequencies across the whole list of k-mers [7,8].

In this study, we identified nucleotide fragments that contain both the genome barcode information and interspecific differences. We then utilized these fragments to perform genomic typing of *mycobacteria*. This study describes a novel tool that can be used to analyze different genomes leading to identification of subtypes of *mycobacteria* and can be implemented for future clinical use or epidemiological studies.

## Materials and methods

### Data on genome sequences of various types of mycobacteria

We downloaded the whole genomic sequences of 17 sequenced *Mycobacterium* strains from the NCBI database (http://www.ncbi.nlm.nih.gov/genome/) in January 2013. These data were used to construct DNA barcoding analyses.

### Calculation of genomic barcode distance

To generate DNA barcoding, we utilized the array matrix of microbiological genomic k-*mer* nucleotide strings and used the Euclidean distance to represent the barcode distance. For example, for any two array matrix M_1_ and M_2_, the computational formula of barcode was as shown below where L was the line and K was the column [9].

$$\sqrt{\overset{L}{\underset{i=1}{\sum }}\overset{K}{\underset{j=1}{\sum }}{\left(M_{1}\left(i,j\right)-M_{2}\left(i,j\right)\right)}^{2}}$$

### Genomic barcode sectionalized identification method

We utilized CLUMP program [10] to cluster the DNA fragments based on barcode similarity. We first selected the DNA fragments randomly as seed-sets on the basis of the density distribution of clustering. We then ran the K-means algorithm. By doing so, we contra-positioned every constructed seed-set and di-clustered multiple times, and then calculated every seed-set by the K-means algorithm [9]. After that, we selected the calculated predicted results of all seed-sets to proceed with the optimized threshold disposal. The computational formula used is shown below:

$$\overset{K}{\underset{i=1}{\sum }}\underset{X\in \mathrm{Ci}}{\sum }{\left(X-\overset{¯}{\mathrm{Xi}}\right)}^{2}$$

### Genotyping of mycobacteria

We first performed a blast search of the three screened genes using the NCBI blast tool (http://blast.ncbi.nlm.nih.gov/) E value set as 0 and the Max index value as ≥ 91%. We then utilized the ClustalX, jModelTest and MEGA (version 5.05) software s to molecularly type different types of *mycobacterium*.

### Functional analysis of barcode genes via Pfam_Scan and Blast2GO

To functionally analyze the barcode genes, we first downloaded Pfam database version 23.0 from ftp://ftp.sanger.ac.uk/pub/databases/Pfam. The Pfam database mainly includes two parts, Pfam_ls and Pfam_fs. In this study, we mostly used the Pfam_ls component. Following this, we switched to the Pfam directory and ran hmmfam program to input the sequence data. Next, we analyzed the sequences through a GO annotating and functional analysis technology, $ hmmpfam --cpu 4 -E 0.0001 Pfam_ls InputSeq.fas > OutResults.fasBLAST2GO. We also used an online software Blast2GO (http://www.blast2go.de/) to annotate the genes, and set the E value as ≤ -10.

## Results

### Genome barcode visual annotation of Mycobacterium

**Figure 1:**
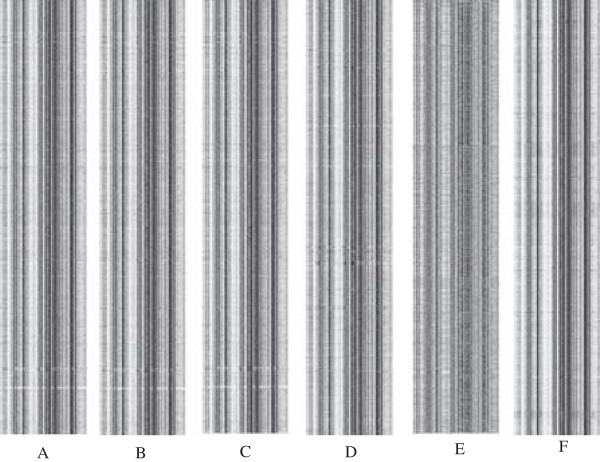
**Genomic barcodes of six *Mycobacterium *species. (A)***M. tuberculosis* H37Rv, **(B)***M. tuberculosis* CDC 1551, **(C)***M. tuberculosis* F11, **(D)***M. marinum*, **(E)***M. leprae* TN and **(F)***M. avium* 104. For each barcode, the X-axis lists 136 unique combinations of 4-mers and their reverse complements arranged in alphabetical order, while the Y-axis is the gray scale value of co-linear with the 4-mer frequencies within the 1000-bp non-overlapping sliding windows along each chromosome.

We first downloaded 17 different *mycobacterium* genome sequences and partitioned them into a plurality of non-overlapping 1000 bp sized fragments. The frequency of nucleotide strings (K-*mer*, K = 4) within the fragments was then calculated. The number of nucleotide strings was set as N (4), N (4) = 128; we thus obtained an array with N (4) as the column and genome length/1000 as the row. Barcode-like annotation was obtained by further processing and transformation of this array to gray-scale images, in which the brighter gray represents the higher frequency, and the dimmer gray represents the lower frequency. The genome barcode visual annotation of *M. tuberculosis H37Rv*, *M. marinum*, *M. leprae TN* and *M. avium 104* is shown in Figure 1. Each *mycobacterium* strain is illustrated as a unique barcode image. These barcode images reflect the genomic DNA component characteristics and enable further identification of DNA fragments containing fractal features.

### Screening of DNA barcoding genes base using distance of genomic barcode

From the visualized *mycobacterium* genome barcode, we were able to find a characteristic region at the bottom of the *M. tuberculosis H37Rv* genome barcode. This region contained two bright stripes, which are specific for *M.* tu*berculosis* compared to NTM. We further compared genetic barcodes of *M. tuberculosis, M. tuberculosis* CDC 1551 and *M. tuberculosis* F11 show that the gene barcodes were almost identical between *M. tuberculosis* H37Rv, *M. tuberculosis* CDC 1551 and *M. tuberculosis* F11. It is evident that closely related strains have a similar barcode. We therefore extracted data from these regions for further analysis. The gene barcode distance can be represented by the calculated Euclidean distance between the frequency of average 4-mer strings in the genome and that of any gene 4-mer strings. Furthermore, the difference in fragment lengths between each genome can be characterized by comparison with the Euclidean distance. Through the calculation of the Euclidean distance of different fragments, three highly polymorphic genes related to pathogenicity of MTB were identified in *M. tuberculosis* H37Rv (named as Rv0279c, Rv3508 and Rv3514) (See Table 1).

**Table 1:** Barcoding genes in the MTB H37Rv genome

**Gene name**	**ID**	**Location**	**Length**	**Function**
Rv0279c	886621	336560-339073	2513 bp	PE-PGRS family protein
Rv3508	888270	3931005-3936710	5705 bp	PE-PGRS family protein
Rv3514	888294	3945794-3950263	4469 bp	PE-PGRS family protein

### Phylogenetic analysis of mycobacteria based on barcoding genes

**Figure 2:**
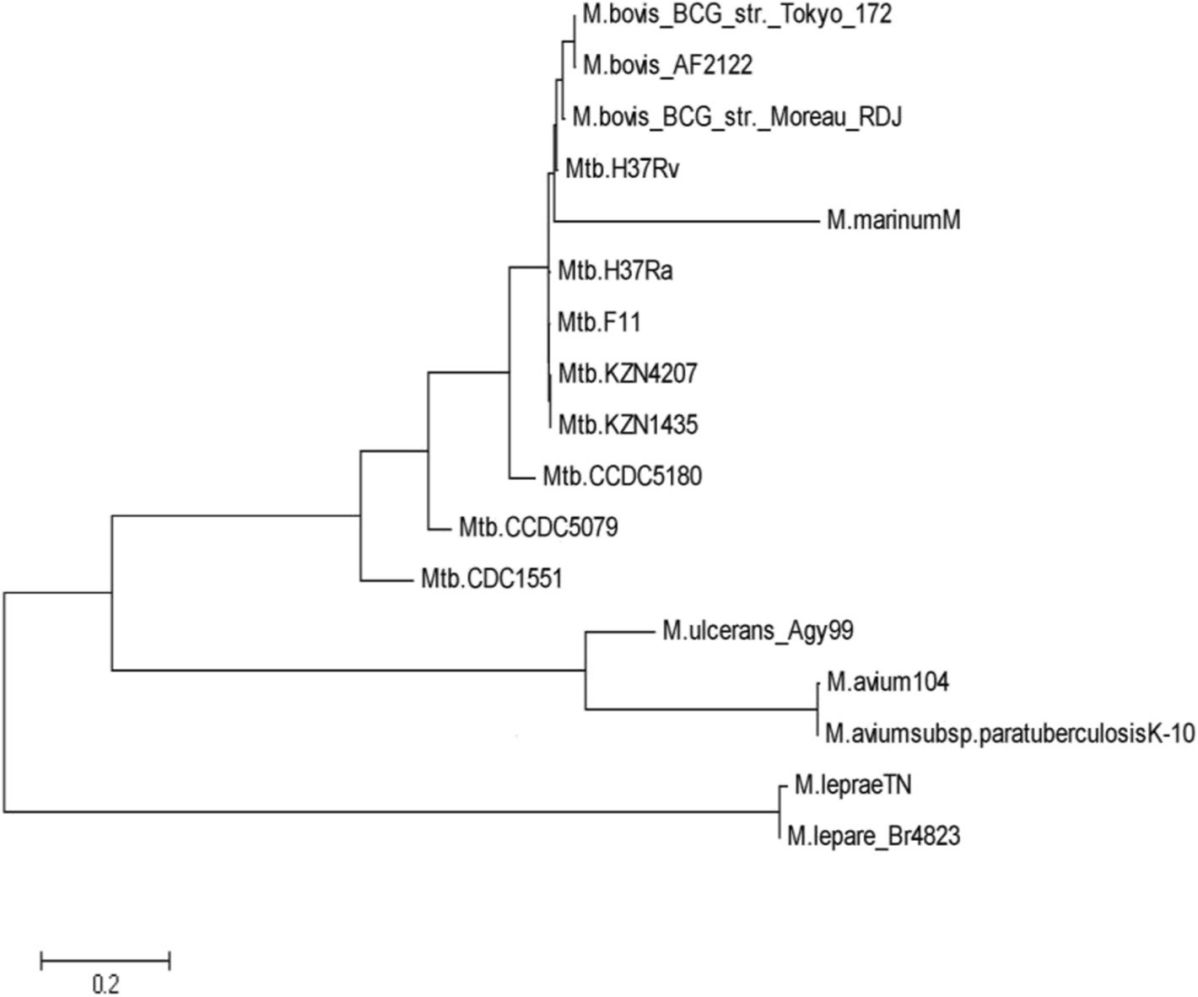
**Phylogenetic analysis of Mycobacterium based on the three barcoding genes.** By utilizing genomic barcodes, we can phylogenetically analyze these different subtypes of *Mycobacteria*.

Utilizing genomic barcodes, we can phylogenetically analyze the different subtypes of *mycobacteria*. The 16S rRNA is a highly conserved and most common gene in the bacterial genome [11,12]. However, methods that involve systematic analysis utilizing 16S rRNA alone may not be enough for phylogenetic analysis. We therefore utilized 16S rRNA combined with the screened barcode genes to analyze *mycobacterium* evolution (phylogenies). Splicing of *Rv0279c*, *Rv3508*, and *Rv3514* with16S rRNA sequences into a long tandem sequences was performed and the barcode distance between two *mycobacterium* genomes was calculated (see Materials and methods section for detail). The pair-wise distance of all the genomes under consideration were entered into the MEGA meg file to build the phylogenetic tree using neighbor-joining method with MEGA 5.05 software (Figure 2). Our results suggest that barcode genes were a good representation of the whole genomic information, which could be useful for molecular typing of *mycobacterium* and for distinguishing between NTM and *mycobacterium*.

## Discussion

In the current study, we have generated a genomic barcode system using genome visualization technology and based on calculation of the base composition of *mycobacterium* genomes. We then identified three genes from *mycobacterium* genomes that have utility in genotyping *mycobacteria.* All of these three genes encoded proteins belonging to the PE-PGRS family, which is unique to MTB. Previous studies showed that single-nucleotide polymorphisms (SNPs) of most MTB genes occurred in the genomic region of the PE/PPE family [13,14]. Functional analysis using Pfam database showed that Rv0279c participates in regulation of iron metabolism in the host [15,16] while Rv3508 participates in oxidative stress [17,18] and Rv3514 is a member of the cellular surface/secreted protein ESX family [19]. These three genes are highly polymorphic and closely associated with the pathogenicity of MTB.

Evolutionary comparison between the various mycobacterial isolates revealed that the genetic distance between *M. tuberculosis* H37Rv and *M. bovis* BCG vaccine was quite close and therefore provides an explanation for the protective effects of *M. bovis* BCG vaccine. The genetic distance between Beijing strain CCDC5079, CCDC5180 and *M. bovis* BCG vaccine was relatively far. The two Beijing strains were isolated from tuberculosis patients in China in 2004 and are the main pandemic strains in China and other Asian countries, such as Japan, Korea, and India [20]. The World Health Organization (WHO) reported that the protective rate of *M. bovis* BCG vaccine in North America and Northern Europe was among the highest (60%-80%), whereas there was no protective effect in the south of India (0%) because the pandemic strains in south of India was the Beijing strain. It is clear that our genomic barcode system can provide information on mycobacteria that is of biological significance and could help with the development of an effective vaccine.

The molecular phylogeny of *mycobacterium* showed that many NTMs have a close genetic distance with MTB. For example, the phylogenetic distance between *M. marinum* and MTB was very short, whereas the phylogenetic distance from *M. avium* was relatively long, which was confirmed by the whole genome sequence alignment analyses [21-23]. Our data showed that *M. marinum* and MTB had a closely genetic relationship with about 3000 homologous fragments between the two strains in addition to the amino acid being 85% (on average) identical. It is possible that the large genome of *M. marinum* allows it to adapt well to the environment and also enables this strain to be more pathogenic to a wide range of hosts. The nature and histologic characteristics of disease caused by *M. marinum* is surprisingly similar to that of MTB. This could be due to *M. marinum* possessing the same set of virulent genes as MTB [24,25]. In conclusion, we propose that our novel gene barcode system is a useful tool in the molecular phylogenetic typing of *mycobacteria.*

## Conclusion

In this report, we built a genomic barcode visualization technology through calculating the base composition of *Mycobacterium*, and screened three genes (Rv0279c, Rv3508 and Rv3514) from the PE/PPE family of MT Band which could be used in *Mycobacterium* typing. These three genes contained the whole genetic information of *Mycobacterium*, which had high distinguishability and combined with 16S rRNA gene could achieve accurate molecular typing. In the future, our genotyping research will support the genetic potentials accurately, and brings hope for conquer disease caused by mycobacterium.

## Competing interests

The authors have declared that no competing interests exist.

## Authors’ contributions

BL and HL conceived the experiments, XZ analyzed the data, YZ contributed reagents/materials/analysis tools, BL wrote the paper, GW and FZ designed the study. All authors read and approved the final manuscript.
